# Healthcare risk stratification model for emergency departments based on drugs, income and comorbidities: the DICER-score

**DOI:** 10.1186/s12873-024-00946-7

**Published:** 2024-02-14

**Authors:** Jesús Ruiz-Ramos, Emili Vela, David Monterde, Marta Blazquez-Andion, Mireia Puig-Campmany, Jordi Piera-Jiménez, Gerard Carot, Ana María Juanes-Borrego

**Affiliations:** 1 Pharmacy Department, Hospital Santa Creu i Sant Pau. Institut de Recerca Sant Pau (IR SANT PAU), Barcelona, Spain; 2 Catalan Health Service. Digitalization for the Sustainability of the Healthcare System (DS3). Institut d’Investigacions Biomèdiques de Bellvitge (IDIBELL), Barcelona, Spain; 3 Catalan Institute of Health, Digitalization for the Sustainability of the Healthcare System (DS3), Institut d’Investigacions Biomèdiques de Bellvitge (IDIBELL), Barcelona, Spain; 4 Emergency Department, Hospital Santa Creu i Sant Pau, Institut de Recerca Sant Pau (IR SANT PAU), Barcelona, Spain

**Keywords:** Polypharmacy, Elderly, Emergency care

## Abstract

**Background:**

During the last decade, the progressive increase in age and associated chronic comorbidities and polypharmacy. However, assessments of the risk of emergency department (ED) revisiting published to date often neglect patients’ pharmacotherapy plans, thus overseeing the Drug-related problems (DRP) risks associated with the therapy burden. The aim of this study is to develop a predictive model for ED revisit, hospital admission, and mortality based on patient’s characteristics and pharmacotherapy.

**Methods:**

Retrospective cohort study including adult patients visited in the ED (triage 1, 2, or 3) of multiple hospitals in Catalonia (Spain) during 2019. The primary endpoint was a composite of ED visits, hospital admission, or mortality 30 days after ED discharge. The study population was randomly split into a model development (60%) and validation (40%) datasets. The model included age, sex, income level, comorbidity burden, measured with the Adjusted Morbidity Groups (GMA), and number of medications. Forty-four medication groups, associated with medication-related health problems, were assessed using ATC codes. To assess the performance of the different variables, logistic regression was used to build multivariate models for ED revisits. The models were created using a “stepwise-forward” approach based on the Bayesian Information Criterion (BIC). Area under the curve of the receiving operating characteristics (AUCROC) curve for the primary endpoint was calculated.

**Results:**

851.649 patients were included; 134.560 (15.8%) revisited the ED within 30 days from discharge, 15.2% were hospitalized and 9.1% died within 30 days from discharge. Four factors (sex, age, GMA, and income level) and 30 ATC groups were identified as risk factors and combined into a final score. The model showed an AUCROC values of 0.720 (95%CI:0.718–0.721) in the development cohort and 0.719 (95%CI.0.717–0.721) in the validation cohort. Three risk categories were generated, with the following scores and estimated risks: low risk: 18.3%; intermediate risk: 40.0%; and high risk: 62.6%.

**Conclusion:**

The DICER score allows identifying patients at high risk for ED revisit within 30 days based on sociodemographic, clinical, and pharmacotherapeutic characteristics, being a valuable tool to prioritize interventions on discharge.

## Highlights

Risk scores are often used to predict the clinical outcomes of patients in many healthcare settings.

To the date, no prediction model of emergency department (ED) visits based on patients’ pharmacotherapy, income level, and comorbidities have been developed.

We have designed an ED risk score combined four risk factors (sex, age, comorbidity score and income level) and 30 drug categories to identify those patients at high risk of health-care visit.

## Introduction

During the last decades, the progressive increase in age and associated chronic comorbidities and polypharmacy in the population has led to a growing demand for healthcare resources, particularly emergency services. Secondary effects of population ageing include the onset of drug-related problems (DRP), which can be due to failure of pharmacotherapy because of ineffectiveness, safety issues, or the need for additional medicines. Approximately 5–10% of the hospitalisations and 10–20% of Emergency Department (ED) visits are due to DRPs, most of them considered avoidable [1–3].

The rise in emergency room visits and unplanned hospital admissions represents one of the primary challenges for healthcare systems globally, particularly in the elderly population. Due to the elevated occurrence of emergencies among older individuals [4], there is a likelihood that older adults will contribute to a growing proportion of visits to ED in the future. Several studies have already highlighted a rising demand for ED services. For instance, in England, the number of ED visits by individuals aged 65 years or older surged by 46% between 2001 and 2012 [5]. In the USA, the annual visit rate among those aged 65 years or older was 511/1000 persons and increased with age [6]. Elderly patients exhibit a higher hospitalization rate, necessitate more resources, and face an elevated risk of adverse outcomes [5]. This increase aligns with the mounting healthcare expenses associated with patient´s progressive and irreversible decline following hospital admissions [4]. Hence, prioritizing the prevention of hospital admissions potentially linked to polypharmacy should be considered a priority for health administrations. Over the past decade, numerous research studies have shown that multidisciplinary initiatives targeting primary prevention of DRP effectively lower the likelihood of ED visits and hospitalizations [7, 8]. However, assessments of the risk of ED revisiting published to date often neglect patients’ pharmacotherapy plans, thus overseeing the DRP risks associated with the therapy burden. Hence, although some algorithms have been developed to predicting (or stratify the risk of) readmissions and health outcomes in patients visited at the ED, none of them combines clinical characteristics (e.g., summary measures of the comorbidity burden), social characteristics, and pharmacotherapy. Considering the high social demand for emergency care, prioritization strategies must be developed for multidisciplinary teams to prevent EDs revisits and hospital readmissions. Several index of frailty status and comorbidity are becoming essential tools in electronic medical records systems, continually expanding in comprehensiveness each year. The availability of tools for risk stratification based on chronic pharmacotherapy will enable the identification of high-risk patients as priorities for optimizing their treatment, offering significant potential to decrease potentially avoidable hospital admissions.

This study aims to create a comprehensive scoring system based on comorbidity burden, social vulnerability, and pharmacotherapy tailored for automated calculation by electronic healthcare systems, facilitating the assessment of short-term risks associated with ED visits and hospital admissions.

## Methods

### Study design, population, and data sources

We designed an observational retrospective population-based study in Catalonia, a North-East region in Spain, with a population of 7.7 million people. The Catalan population receives comprehensive healthcare services from the regional Catalan Health Service, utilizing a network comprising 64 general hospitals, 27 psychiatry hospitals, 375 primary care centers, 91 skilled nursing facilities for intermediate care, and 130 outpatient mental health facilities. Detailed sociodemographic and clinical information, including diagnoses, annual income, and healthcare utilization, has been gathered from the Catalan Health Surveillance System (CHSS) since 2011, encompassing the entire population of Catalonia. This record, used in previous publications in other areas [9–11], gathers data recorded in multiple settings, linked through a unique identification number used for public insurance purposes. These environments encompass primary care, acute care hospitals, intermediate care hospitals, mental health centers, outpatient clinics, and emergency services. Additionally, this documentation compiles data pertaining to prescriptions, pharmacy costs, and invoices, encompassing non-urgent medical transportation, outpatient rehabilitation, home oxygen therapy, and dialysis. No data about private healthcare could be collected because these centers use different codes for patient identification. However, owing to the co-payment system of medicines established in Spain, chronic prescriptions outside the public health system are unusual. Medications are introduced in the registry using the codes of the Anatomical Therapeutic Chemical (ATC) Classification System.

The aforementioned information originates from the interactions between patients and any public healthcare entity or service and is regularly transferred from the electronic health records of healthcare providers to the Catalan Health Service (the public insurer in Catalonia), which uses it for billing purposes, among others.

The study included data from all visits to life-threatening hospital emergencies (triage 1, 2, or 3) between January 1 and December 31, 2019. The following cases were excluded from the analysis: emergencies in children under 18 years of age; emergencies with triage 4 or 5; emergencies without completion of care: transfer or referral to another health center, and evasion or voluntary or administrative discharge; deaths in the emergency department; v) emergencies without patient identifier; emergencies with the main diagnosis of complications of pregnancy, childbirth and postpartum (system 11); and emergencies with the main diagnosis of injuries (CCS 00225, 00240 and 00244) [12].

Once selected the study cohort, the full dataset was randomly split into a model development cohort (60%) and validation cohort (40%).

All data were handled according to the General Data Protection Regulation 2016/679 on data protection and privacy for all individuals within the European Union and the local regulatory framework regarding data protection [13]. Data from different health administrative databases were linked and de-identified by a team not involved in the study analysis; study investigators only had access to a fully anonymized database. The retrospective use of healthcare data was approved by the Hospital Santa Creu i Sant Pau Ethics Committee (Nº: (Nº: IIBSP-COD-2022-40), which waived the need for obtaining informed consent for data utilization. Results are presented according to the Transparent reporting of a multivariable prediction model for individual prognosis or diagnosis (TRIPOD) guidelines. STROBE and RECORD guidelines for observational studies and studies using routinely collected health data were also considered.

### Variables

The sociodemographic variables considered in this study were age, sex, and income level, classified as high (annual income > 100,000 €), intermediate (18,000–100,000 €), low (< 18,000 €), and very low (receiving welfare support from the government) [14, 15]. Comorbidity burden was calculated based on the Adjusted Morbidity Groups (GMA) [16–18]. The GMA tool considers all chronic diagnoses present at a given time and acute diagnoses reported during the study period. The GMA index score is computed by adding the weights of each diagnosis group, being used to generate mutually-exclusive risk groups based on the index distribution in the general population as follows: baseline risk (healthy stage; GMA index up to the 50th percentile of the total population), low risk (GMA index between the 50th and 80th percentiles), moderate risk (GMA risk between the 80th and 95th percentiles), high risk (GMA index between the 95th and 98th percentiles), and very-high risk (GMA index above the 98th percentile). Forty-four medication groups associated with medication-related health problems were assessed using ATC codes [19] dispensed in the two months preceding the index visit to the emergency room. The number of different drugs (5-digit ATC) dispensed during the previous two months is also calculated. This last measure was analyzed as a continuous variable.

### Endpoints

Study outcomes included all-cause revisiting to the ED, hospital admission, and death within the 30 days following discharge of the index episode. The primary outcome, used as a response variable for model development and validation, was a composite of achieving any of the three outcomes.

### Statistics

Categorical variables were described as frequencies and percentages, and quantitative variables as the mean and standard deviation (SD). Categorical variables were compared using Pearson’s Chi-squared test with Yates’ continuity correction. The statistical significance threshold was set at a bilateral alpha value of 0.05.

The dataset’s composition was driven by events; all included factors were either clinical conditions or variables essential for healthcare system registration. Consequently, the variables analyzed contained no missing data, and no efforts were made to fill in any gaps through data imputation.

To assess the performance of the different variables, we used generalized linear models (logistic regression) to build multivariate models for ED revisits, with the contribution of each factor expressed as an odds ratio (OR) and its 95% confidence interval (CI). The models were created using a “stepwise-forward” approach based on the Bayesian Information Criterion (BIC), in which a naïve model is sequentially complemented with the most relevant variables, eventually leading to the main effects model [20].

In both the development and validation datasets, we evaluated the performance of each model using various statistical measures. For our primary analysis, we opted for the area under the curve of the receiving operating characteristics (AUCROC) curve. This curve assesses the model’s discriminatory ability as the threshold varies, ranging from 0.5 (indicating low discrimination capacity) to 1 (indicating high discrimination capacity). AUC-ROCs of < 0.70, 0.70–0.89, and ≥ 0.90 were considered poor, adequate, and excellent, respectively. Furthermore, we conducted secondary analyses employing the Bayesian Information Criterion (BIC) and the area under the precision-recall (AUC-PR) curve. The BIC quantifies in-sample prediction error, considering the trade-off between achieving a good fit (avoiding overfitting) and maintaining model simplicity (preventing underfitting). The range of values for the BIC is contingent on the study sample, with lower values denoting superior performance and higher values indicating poorer performance. On the other hand, the AUC-PR curve delineates the balance between precision (minimizing false-positive rates) and recall (minimizing false-negative rates). Unlike the ROC curve, the AUC-PR curve provides a less biased assessment, especially for outcomes with low frequency [21]. All analyses were conducted using the R statistical package, version 4.0.3 [22].

### Ethics approval

The study protocol was approved by the Independent Institutional Review Board of the Hospital Santa Creu I Sant Pau Ethics Committee, which waived the need for written informed consent (protocol code IIBSP-PRM-2021-39 (PI21/01818).

The results obtained are presented in accordance with the Transparent reporting of a multivariable prediction model for individual prognosis or diagnosis (TRIPOD) guidelines. This study adhered to the Declaration of Helsinki guidelines.

## Results

### Study cohort

The study group included 834,679 cases with a mean age of 49.1 (SD: 18.2 years). The main characteristics of the study cohort are shown in Table 1. Overall, 271,270 (32.5%) individuals met any of the primary endpoint criteria: 131,879 (15.8%) had ED revisits, 126,871 (15.2%) hospital readmissions, and 75,956 (9.1%) died within the 30 days following discharge from ED. The frequency of the composite endpoint was higher among men, increased with the number of drugs prescribed, and decreased with the income level.

**Table 1:** 30-day composite endpoint of the six outcome variables considered in the analysis

	Total *N* = 851,649	Development *N* = 510,989	Validation *N* = 340,660	p-value
Sex				0.181
Men	409,746 (48.1%)	245,545 (48.1%)	164,201 (48.2%)	
Women	441,903 (51.9%)	265,444 (51.9%)	176,459 (51.8%)	
Age	60.0 (20.9)	60.0 (20.9)	60.1 (20.9)	0.156
Age group				0.762
18–34	125,493 (14.7%)	75,328 (14.7%)	50,165 (14.7%)	
35–44	104,704 (12.3%)	62,953 (12.3%)	41,751 (12.3%)	
45–54	110,938 (13.0%)	66,775 (13.1%)	44,163 (13.0%)	
55–64	112,484 (13.2%)	67,455 (13.2%)	45,029 (13.2%)	
65–74	133,879 (15.7%)	80,298 (15.7%)	53,581 (15.7%)	
75–84	150,794 (17.7%)	90,347 (17.7%)	60,447 (17.7%)	
84–94	104,504 (12.3%)	62,533 (12.2%)	41,971 (12.3%)	
>94	8853 (1.04%)	5300 (1.04%)	3553 (1.04%)	
Income level				0.130
High	3434 (0.40%)	2110 (0.41%)	1324 (0.39%)	
Medium	203,093 (23.8%)	121,749 (23.8%)	81,344 (23.9%)	
Low	594,152 (69.8%)	356,379 (69.7%)	237,773 (69.8%)	
Very Low	50,970 (5.98%)	30,751 (6.02%)	20,219 (5.94%)	
GMA index score				0.543
Baseline risk	68,083 (7.99%)	41,061 (8.04%)	27,022 (7.93%)	
Low risk	195,580 (23.0%)	117,297 (23.0%)	78,283 (23.0%)	
Moderate risk	281,024 (33.0%)	168,575 (33.0%)	112,449 (33.0%)	
High risk	203,696 (23.9%)	122,101 (23.9%)	81,595 (24.0%)	
Very high risk	103,266 (12.1%)	61,955 (12.1%)	41,311 (12.1%)	
Number of drugs	6.14 (4.72)	6.14 (4.73)	6.15 (4.72)	0.614
Antiacids (A02)	351,793 (41.3%)	211,112 (41.3%)	140,681 (41.3%)	0.872
Osmotic Laxatives (A06AD)	2279 (0.27%)	1376 (0.27%)	903 (0.27%)	0.729
Other Laxatives (A06X)	2524 (0.30%)	1498 (0.29%)	1026 (0.30%)	0.518
Insulin (A10A)	53,248 (6.25%)	31,908 (6.24%)	21,340 (6.26%)	0.713
Sulfonylides (A10BB)	19,334 (2.27%)	11,622 (2.27%)	7712 (2.26%)	0.754
Gliptines (A10BH)	21,370 (2.51%)	12,925 (2.53%)	8445 (2.48%)	0.147
Glyphosines and glinides (A10BX)	10,029 (1.18%)	6062 (1.19%)	3967 (1.16%)	0.366
Other Oral antidiabetics (A10BX)	71,463 (8.39%)	42,778 (8.37%)	28,685 (8.42%)	0.428
Vitamin K Antagonists (B01AA)	52,971 (6.22%)	31,713 (6.21%)	21,258 (6.24%)	0.527
Heparin (B01AB)	37,724 (4.43%)	22,635 (4.43%)	15,089 (4.43%)	0.999
Antiplatelet (B01AC)	154,251 (18.1%)	92,367 (18.1%)	61,884 (18.2%)	0.293
Other antithrombotic (B01AX)	33,035 (3.88%)	19,891 (3.89%)	13,144 (3.86%)	0.426
Digoxin (C01AA)	11,668 (1.37%)	6992 (1.37%)	4676 (1.37%)	0.875
Antiarrhythmics (C01B)	19,508 (2.29%)	11,745 (2.30%)	7763 (2.28%)	0.557
Antihypertensives (C02)	20,922 (2.46%)	12,474 (2.44%)	8448 (2.48%)	0.261
Potassium Saving Agents (C03D)	26,609 (3.12%)	16,001 (3.13%)	10,608 (3.11%)	0.655
Other diuretics (C03X)	140,610 (16.5%)	84,188 (16.5%)	56,422 (16.6%)	0.290
Beta blockers (C07A)	143,490 (16.8%)	85,989 (16.8%)	57,501 (16.9%)	0.537
Verapamil or diltiazem (C08D)	13,575 (1.59%)	8240 (1.61%)	5335 (1.57%)	0.095
Other calcium antagonists (C08X)	85,654 (10.1%)	51,164 (10.0%)	34,490 (10.1%)	0.094
IECA / ARA-II (C09)	257,782 (30.3%)	154,414 (30.2%)	103,368 (30.3%)	0.220
Statins (C10AA)	195,564 (23.0%)	116,962 (22.9%)	78,602 (23.1%)	0.048
Fibrates (C10AB)	13,480 (1.58%)	8156 (1.60%)	5324 (1.56%)	0.232
Cotrimoxazole (D01AC01)	4970 (0.58%)	2958 (0.58%)	2012 (0.59%)	0.495
Systemic corticosteroids (H02)	115,691 (13.6%)	69,158 (13.5%)	46,533 (13.7%)	0.098
Beta-lactam antibiotics (J01DH)	1162 (0.14%)	693 (0.14%)	469 (0.14%)	0.825
Other antibiotics (J01X)	268,849 (31.6%)	161,296 (31.6%)	107,553 (31.6%)	0.952
Folic Acid Analogs (L01BA)	944 (0.11%)	579 (0.11%)	365 (0.11%)	0.421
Tacrolimus (L04AD02)	5574 (0.65%)	3287 (0.64%)	2287 (0.67%)	0.119
Other calcineurin inhibitors (L04AD)	690 (0.08%)	414 (0.08%)	276 (0.08%)	1.000
Other immunosuppressants (L04X)	13,768 (1.62%)	8279 (1.62%)	5489 (1.61%)	0.756
Anti-inflammatory (M01)	181,636 (21.3%)	108,899 (21.3%)	72,737 (21.4%)	0.658
Opiacis (N02A)	135,176 (15.9%)	80,875 (15.8%)	54,301 (15.9%)	0.164
Pyrazolones (N02BB)	137,802 (16.2%)	82,552 (16.2%)	55,250 (16.2%)	0.440
Phenytoin (N03AB02)	1165 (0.14%)	678 (0.13%)	487 (0.14%)	0.220
Carboxamide (N03AF)	7606 (0.89%)	4540 (0.89%)	3066 (0.90%)	0.587
Valproic acid (N03AG01)	7452 (0.88%)	4449 (0.87%)	3003 (0.88%)	0.606
Other antiepileptics (N03X)	86,398 (10.1%)	51,789 (10.1%)	34,609 (10.2%)	0.718
Liti (N05AN01)	2180 (0.26%)	1342 (0.26%)	838 (0.25%)	0.143
Other antipsychotics (N05AX)	73,728 (8.66%)	44,342 (8.68%)	29,386 (8.63%)	0.410
Benzodiazepines (N05BA)	180,127 (21.2%)	108,302 (21.2%)	71,825 (21.1%)	0.222
Other psycholeptics (N05X)	36,030 (4.23%)	21,563 (4.22%)	14,467 (4.25%)	0.549
IRS Antidepressants (N06AB)	105,143 (12.3%)	63,293 (12.4%)	41,850 (12.3%)	0.164
Other psychoanaleptics (N06AX)	72,004 (8.45%)	43,118 (8.44%)	28,886 (8.48%)	0.505

### Model development and validation

**Fig. 1:**
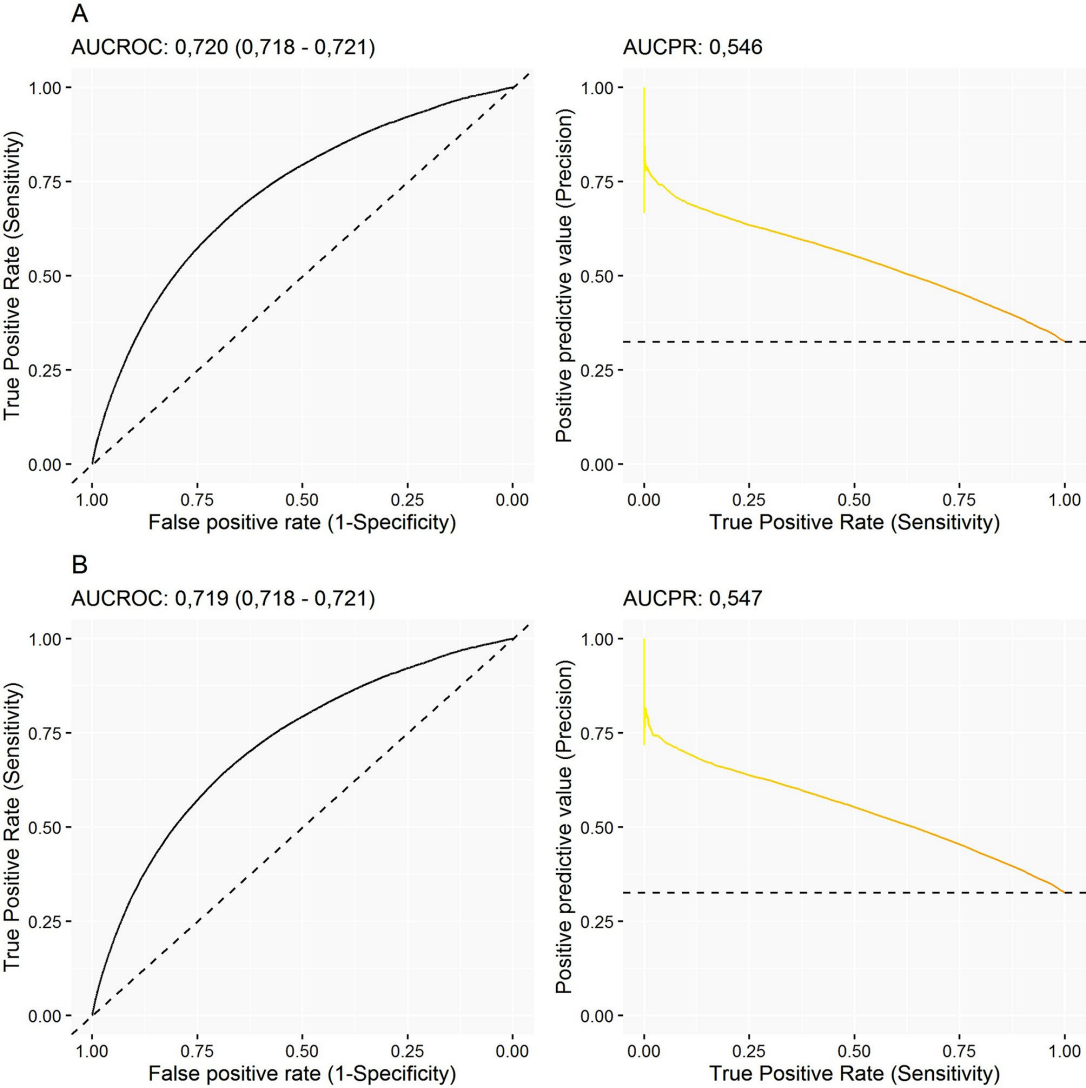
Performance of the DICER score for predicting the composite endpoint of ED revisit, hospital admission or death within the 30 days following discharge from the index episode. (**A**): development dataset. (**B**): validation dataset. AUCROC: area under the curve of the receiving operating characteristics. AUCPR: area under the precision-recall curve

**Fig. 2:**
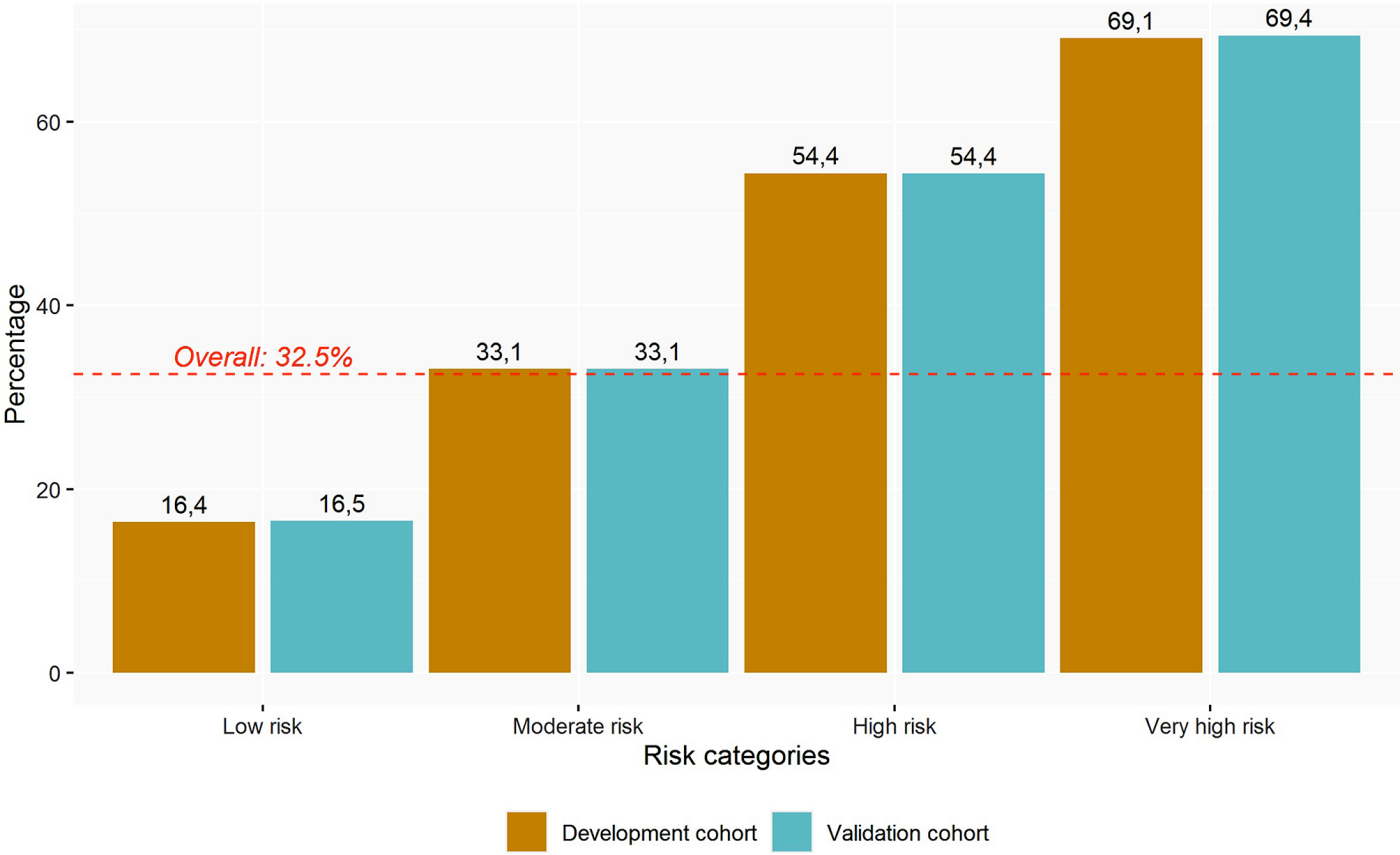
Risk of 30-day composite variable (ED revisit, hospital admission or death) for the predictive and validation population according to the four established risk groups

Table 2 summarizes the contribution of each parameter (sex, age, income level, GMA index, and drug dispensation, classified by ATC groups) to explaining the composite endpoint. The DICER-Score value is obtained after incorporating each of the beta coefficients into the logistic regression model [Risk for ED visit: 1/(1-exp(Constant+$\sum xbeta$)].The assessment of the comorbidity burden using the GMA as a summary measure accounted for the highest effect size on the composite endpoint. The final model (DICER-Score) reached an AUROC of 0.720 (95%CI 0.718–0.721) in the development sample and 0.719 (95%CI 0.718–0.721) in the validation sample (Fig. 1), consistently with an adequate convergent validity. Four risk categories were established: low risk (Risk for ED visit cut off-value: 16.4%), moderate risk (Risk for ED visit: 33.1%), high risk (Risk for ED visit: 54.4%) and very high risk (Risk for ED visit: 69.1%). Figure 2 shows the agreement between the development and validation cohort regarding the frequency of the composite endpoint of ED revisit, hospital re-admission or death within the 30 days following ED discharge.

**Table 2:** Relative Risk of 30-day Emergency Department visits for the variables included in the multivariable analysis

	n	%	Coeff. (beta)	Odds ratio	IC95%
Sex					
Men	245.545	35,4%	0	1	
Women	265.444	29,8%	-0,25	0.765	0.755–0.775
Age					
18–34	75.328	18,1%	0	1	
35–44	62.953	19,3%	-0,05	0.949	0.922–0.975
45–54	66.775	22,6%	-0,05	0.956	0.930–0.983
55–64	67.455	30,5%	0,15	1.152	1.120–1.184
65–74	80.298	37,0%	0,22	1.248	1.213–1.284
75–84	90.347	43,4%	0,33	1.389	1.350–1.430
84–94	62.533	51,9%	0,62	1.864	1.808–1.922
>94	5.300	59,1%	1.00	2.702	2.539–2.875
Risk stratification (GMA):					
Baseline risk	41.061	12,6%	0	1	
Low risk	117.297	17,5%	0,35	1.417	1.371–1.465
Moderate risk	168.575	27,9%	0,75	2.107	2.037–2.179
High risk	122.101	44,8%	1,20	3.329	3.209–3.453
Very high risk	61.955	62,1%	1,71	5.537	5.317–5.766
Income level					
High	2.110	29,5%	0		
Intermediate	121.749	29,4%	-0,09	0.916	0.828–1.013
Low	356.379	33,4%	-0,03	0.970	0.878–1.073
Very low	30.751	34,2%	0,05	1.055	0.952–1.170
Number of drugs			0,04	1,038	1,034 − 1,041
ATC groups					
Antiacids (A02)	211.112	42,0%	0,04	1.041	1,025 − 1,058
Osmotic Laxatives (A06AD)	1.376	65,1%	-0,12	1.580	1.407–1.776
Other Laxatives (A06X)	1.498	42,5%	0,46	0.886	0.794–0.988
Sulfonylureas (A10BB)	11.622	38,8%	-0,06	0.874	0.839–0.911
Gliptines (A10BH)	12.925	48,6%	-0,13	0.936	0.901–0.973
Glyphosines and glinides (A10BX)	6.062	48,6%	-0,07	0.947	0.896–1.000
Other Oral antidiabetics (A10BX)	42.778	41,3%	-0,05	0.938	0.917–0.960
Vitamin K Antagonists (B01AA)	31.713	46,8%	-0,20	0.858	0.834–0.882
Heparin (B01AB)	22.635	50,0%	-0,15	1.446	1.404–1.490
Antiplatelet (B01AC)	92.367	44,6%	0,37	0.985	0.966–1.004
Other antithrombotic (B01AX)	19.891	45,2%	-0,02	0.815	0.788–0.843
Digoxin (C01AA)	6.992	52,6%	0,06	1.058	1.005–1.114
Antiarrhythmics (C01B)	11.745	44,9%	0,04	1.039	0.997–1.082
Potassium Saving Agents (C03D)	16.001	57,0%	0,08	1.349	1.301–1.399
Other diuretics (C03X)	84.188	49,4%	0,30	1.085	1.065–1.106
Beta blockers (C07A)	85.989	45,2%	0,02	1.024	1.005–1.044
Verapamil or diltiazem (C08D)	8.240	46,3%	-0,07	0.943	0.899–0.988
Other calcium antagonists (C08X)	51.164	42,7%	-0,06	0.928	0.909–0.948
ACEI / ARB-II (C09)	154.414	39,0%	-0,19	0.831	0.818–0.844
Statins (C10AA)	116.962	40,5%	-0,20	0.820	0.806–0.835
Fibrates (C10AB)	8.156	35,5%	-0,18	0.832	0.792–0.873
Systemic corticosteroids (H02)	69.158	49,0%	0,36	1.437	1.409–1.465
Beta-lactam antibiotics (J01DH)	693	64,9%	0,16	2.053	1.740–2.422
Other antibiotics (J01X)	161.296	41,2%	0,72	1.178	1.160–1.196
Folic Acid Analogs (L01BA)	579	75,3%	1,00	2.719	2.234–3.309
Tacrolimus (L04AD02)	3.287	47,3%	-0,19	0.706	0.656–0.761
Other calcineurin inhibitors (L04ADX)	414	40,1%	-0,29	0.750	0.609–0.925
Other immunosuppressants (L04X)	8.279	37,5%	-0,35	0.826	0.787–0.867
Anti-inflammatory (M01)	108.899	26,1%	-0,15	0.865	0.850–0.881
Opioids (N02A)	80.875	42,4%	0,10	1.108	1.088–1.128
Pyrazolones (N02BB)	82.552	36,2%	0,06	1.065	1.046–1.085
Carboxamide (N03AF)	4.540	34,3%	-0,04	0.929	0.870–0.992
Other antiepileptics (N03X)	51.789	40,3%	-0,07	0.958	0.938–0.979
Lithium (N05AN01)	1.342	33,8%	-0,07	1.206	1.072–1.357
Other antipsychotics (N05AX)	44.342	45,1%	0,17	1.180	1.154–1.207
Benzodiazepines (N05BA)	108.302	37,0%	0,19	0.929	0.913–0.944
Other psycholeptics (N05X)	21.563	41,4%	-0,07	0.931	0.903–0.960
IRS antidepressants (N06AB)	63.293	38,3%	-0,10	0.872	0.854–0.890
Other psychoanaleptics (N06AX)	43.118	40,4%	-0,14	0.907	0.886–0.929
Constant			-1,86		

## Discussion

Our results show that a comprehensive view of the patient context, including demographic characteristics, comorbidity burden, socioeconomic status, and drug dispensation has robust predictive capacity of the risk for ED revisit and hospital admission at ED discharge. Although the comorbidity burden has a strong effect in our model, the number and type of drugs dispensed significantly contributed to explaining this risk. This result indicates that drug consumption, which is typically disregarded in risk models at the ED, shall be considered when appraising the likelihood of a patient to revisit or be admitted to a hospital.

Risk scores are often used to predict the clinical outcomes of patients in many healthcare settings [23, 24]. In the context of pharmacotherapy, several scales have been developed for predicting adverse effects in hospitalized patients [25, 26]. In many of them, the risk factors identified are similar to those in our score, including the patient´s age, comorbidity scales, or anticoagulation. In the field of ED, Hao et al. [8]. developed a model for predicting ED revisits based on multiple variables, obtaining a prospective prediction value of 0.704, similar than the obtained in our model. Other studies, which have formulated risk scales to forecast emergency visits but concentrated on specific pathologies such as cancer [27] or decompensated heart failure [28], have yielded predictive values comparable to those observed in our investigation, falling within the range of 0.70 to 0.80. However, to date, no specific revisit prediction scales based on patients’ pharmacotherapy, income level, and comorbidities have been developed, being this point the main strength of this score. As previous risk scores published [16–18], the model’s incorporation of a multitude of variables renders this score valuable for its automated calculation by electronic medical information systems. Our score highlights that individuals with high chronic disease (and consequent therapeutic) burden are particularly prone to revisiting the ED, in line with previous studies [4, 29]. The results of our score are intended to be helpful for prioritizing patients who are candidates for interventions to improve their pharmacotherapy plan in a highly frequented area with a large number of annual visits, such as the ED. Our analysis serves as an illustration of how proficient information systems can facilitate the creation of “learning healthcare systems.” These systems strive to enhance the quality of care by iteratively embracing and evaluating evidence-based solutions.

Osmotic laxatives, β-lactamic antibiotics, and folic acid analogs were the three therapeutic groups associated with a higher risk of ED revisits or hospitalizations. Constipation is a well-known cause of ED visits, particularly in older patients, and anticholinergic burden associated with chronic pharmacotherapy has been associated with ED visits [30]. Although previous studies have found an association between anticholinergic burden and hospital care in geriatric patients, results of early treatment optimization after hospital discharge have not yet been reported. Our data indicate the need for the optimization of long-term treatments in this group of patients. Additionally, ED visits associated with antibiotic failure have also been identified as an important issue in the ED [31]. Finally, it is well known that patients with folic acid analogs (mainly methotrexate) have a high risk of hospitalization due to infections or other drug adverse events [32, 33]. Our results indicate that this group of patients must be under special surveillance and followed up after discharge from these units. Other factors such as advanced age, polypharmacy, or low socioeconomic status are common factors associated with the risk of ED visits, adverse effects, and hospital admissions [34, 35].

The development of this score represents a noteworthy advancement in identifying and optimizing patients with polypharmacy and a high risk of re-consultation, with a distinct applicability in both primary care and among patients discharged from emergency services, particularly those consulting for drug-related issues. The complexity of pharmacotherapy and its adverse effects in frail patients is widely recognized as being associated with a higher risk of emergency room visits and hospital admissions. The implementation of this risk scale will enable the creation of specific programs aimed at optimizing pharmacotherapy and providing close monitoring based on the risk of hospital consultation. Multiple interventions have been performed and analyzed to reduce hospital admissions and ED visits secondary to DRPs. Various studies have observed that interventions such as patient education, medication review on discharge, or telephone consultations can reduce admissions to the ED [29, 36]. Ravn-Nielsen et al. and Juanes A et al. demonstrated that interventions based on treatment revision at discharge and over a telephone call could reduce the risk of a revisit [4, 37]. In this regard, the DICER-score provides clinicians with a helpful tool to increase the efficiency of these interventions and optimize resources in the ED to reduce revisit rates.

### Limitations

This study has some limitations that should be considered. First, it was conducted in a specific population within a specific healthcare system. External validation may be needed to extrapolate the results obtained in areas with a different heathcare system. The results of the DICER score include different variables and multiple ATC groups. That is why direct calculation by electronic medical records is desirable for its efficient application. Second, despite the novelty of considering the socioeconomic status in our model, this classification is based on annual income solely and lacks information regarding social support and other vulnerability sources. Nevertheless, the characteristics of the source dataset in terms of the quality and extension should be considered as a strength that provides robustness to the predictive model. It is worth mentioning that, while the DICER score can predict patients at a high risk of ED revisiting or hospitalization, further studies must be conducted to establish the most appropriate strategies to reduce revisits in this population. The AUROC obtained in our model is lower than 0.8, that has been extensively considered as a very good predictor cut-off value [38]. The individual risk of ED visit is highly variable, in which culture and sociodemographic variables not included in medical records are involved. However, the AUCROC value obtained is higher than other commonly used in clinical practice [23, 24]. Finally, the DICER score, as well as other multimorbidity indices, may have limited capacity to predict outcomes in specific populations like children or patients with mental disorders.

Future investigations should undertake the validation of this scale across diverse populations, considering the characteristics of each population, including variations in comorbidities and the pharmacological treatment approaches for chronic conditions. The utilization and adjustment of this scale in conditions characterized by a high incidence of emergency room visits, and the integration of novel drugs into their management (e.g., chronic heart failure), warrant careful evaluation.

Additionally, it is crucial to compare the outcomes derived from this scale with established clinical practice metrics such as the anticholinergic load or fall risk screening scores. Furthermore, there is a need for fresh research to explore whether the modification and simplification of chronic treatment, as reflected in the risk scale reduction, translate into a tangible decrease in emergency room visits. This would contribute valuable insights into the practical implications of using the scale in optimizing patient outcomes and healthcare resource utilization.

## Conclusion

This study underscores the need for using comprehensive approaches to the assessment of risk of undesired outcomes in individuals visited at the ED. The proposed score shows that not only demographic and clinical characteristics but also socioeconomic status and pharmacotherapy account for the risk of ED revisit or hospital admission within the 30 days following discharge. Importantly, the contribution of pharmacotherapy varies across ATC groups. The DICER score may help clinicians and hospital managers identifying patients at higher risk of ED revisiting and hospital admissions, with potential implications not only in the quality of care but also in resource allocation and planning.

## Data Availability

The data that support the findings of this study are available from the corresponding author upon reasonable request.

## References

[1] Laureau M, Vuillot O, Gourhant V, Perier D, Pinzani V, Lohan L (2021). Adverse drug events detected by clinical pharmacists in an Emergency Department: a prospective Monocentric Observational Study. J Patient Saf.

[2] Bos JM, Kalkman GA, Groenewoud H, van den Bemt PMLA, De Smet PAGM, Nagtegaal JE (2018). Prediction of clinically relevant adverse drug events in surgical patients. PLoS ONE.

[3] Patel P, Zed PJ (2002). Drug-related visits to the emergency department: how big is the problem? Pharmacotherapy. julio de.

[4] Ukkonen M, Jämsen E, Zeitlin R, Pauniaho SL (2019). Emergency department visits in older patients: a population-based survey. BMC Emerg Med.

[5] Aminzadeh F, Dalziel WB (2002). Older adults in the emergency department: a systematic review of patterns of use, adverse outcomes, and effectiveness of interventions. Ann Emerg Med.

[6] Albert M, McCaig LF, Ashman JJ (2013). Emergency department visits by persons aged 65 and over: United States, 2009–2010. NCHS data brief, no 130.

[7] Ravn-Nielsen LV, Duckert ML, Lund ML, Henriksen JP, Nielsen ML, Eriksen CS (2018). Effect of an In-Hospital multifaceted clinical pharmacist intervention on the risk of Readmission. JAMA Intern Med.

[8] Hao S, Jin B, Shin AY, Zhao Y, Zhu C, Li Z, et al. Risk prediction of Emergency Department Revisit 30 days Post Discharge: a prospective study. PLoS ONE [Internet]; 2014. 10.1371/journal.pone.0112944 PMC4231082 25393305

[9] Vela E, Carot-Sans G, Clèries M, Monterde D, Acebes X, Comella A (2022). Development and validation of a population-based risk stratification model for severe COVID-19 in the general population. Sci Rep.

[10] Farré N, Vela E, Clèries M, Bustins M, Cainzos-Achirica M, Enjuanes C (2017). Real world heart failure epidemiology and outcome: a population-based analysis of 88,195 patients. PLoS ONE.

[11] Epidemiology of major. osteoporotic fractures: a population-based analysis in Catalonia, Spain - PubMed [Internet]. [cited 18 april 2022]. Available at: https://pubmed.ncbi.nlm.nih.gov/35267128/. 10.1007/s11657-022-01081-1 35267128

[12] Agency for Healthcare Research and Quality. Clinical Classifications Software (CCS) for ICD-9-CM [Internet]. [cited 2020 Oct 29]. Available from: https://www.hcup-us.ahrq.gov/toolssoftware/ccs/ccs.jsp.

[13] European Parliament and Council Regulation (EU). 2016/679 of 27 April 2016 on the protection of natural persons with regard to the processing of personal data and on the free movement of such data, and repealing Directive 95/46/EC (General data Protection Regulation) [2016] OJ L119/1.

[14] Cainzos-Achirica M, Capdevila C, Vela E, Cleries M, Bilal U, Garcia-Altes A (2019). Individual income, mortality and healthcare resource use in patients with chronic heart failure living in a universal healthcare system: a population-based study in Catalonia, Spain. Int J Cardiol.

[15] Bilal U, Cainzos-Achirica M, Cleries M, Santaeugènia S, Corbella X, Comin-Colet J (2019). Socioeconomic status, life expectancy and mortality in a universal healthcare setting: an individual-level analysis of > 6 million Catalan residents. Prev Med.

[16] Monterde D, Vela E, Clèries M (2016). Los grupos de morbilidad ajustados: nuevo agrupador de morbilidad poblacional de utilidad en El ámbito de la atención primaria. Aten Primaria.

[17] Monterde D, Vela E, Clèries M, Garcia-Eroles L, Roca J, Pérez-Sust P (2020). Multimorbidity as a predictor of health service utilization in primary care: a registry-based study of the Catalan population. BMC Fam Pract.

[18] Vela E, Clèries M, Monterde D, Carot-Sans G, Coca M, Valero-Bover D (2021). Performance of quantitative measures of multimorbidity: a population-based retrospective analysis. BMC Public Health.

[19] High-risk drugs in chronic patients. ISMP-Spain. [accessed 20 Mar 2022]. Available in: http://www.ismp-espana.org/ficheros/Relaci%C3%B3n%20medicamentos%20alto%20riesgo%20en%20cronicos.pdf.

[20] Chowdhury MZI, Turin TC (2020). Variable selection strategies and its importance in clinical prediction modelling. Fam Med Community Health.

[21] Saito T, Rehmsmeier M (2015). The precision-recall plot is more informative than the ROC plot when evaluating binary classifiers on imbalanced datasets. PLoS ONE.

[22] R Core Team. R: A language and environment for statistical com-putting [Internet]. R Foundation for Statistical Computing, Vienna, Austria. 2017 [cited 2022 March 25]. Available from: https://www.r-project.org.

[23] Biancari F, Asim Mahar MA, Kangasniemi OP (2013). CHADS2 and CHA2DS2-VASc scores for prediction of immediate and late stroke after coronary artery bypass graft surgery. J Stroke Cerebrovasc Dis off J Natl Stroke Assoc.

[24] Knaus WA, Draper EA, Wagner DP, Zimmerman JE (1985). APACHE II: a severity of disease classification system. Crit Care Med.

[25] Falconer N, Barras M, Cottrell N (2018). Systematic review of predictive risk models for adverse drug events in hospitalized patients. Br J Clin Pharmacol.

[26] Ruiz Ramos J, Gras-Martin L, Juanes Borrego AM, Blazquez-Andion M, Puig Campmany M, Mangues-Bafalluy MA (2021). Development of an emergency revisit score for patients with drug-related problems. J Pharm Technol.

[27] Lee AR, Park H, Yoo A, Kim S, Sunwoo L, Yoo S (2023). Risk prediction of Emergency Department visits in patients with Lung Cancer using machine learning: Retrospective Observational Study. JMIR Med Inf.

[28] Chae S, Davoudi A, Song J, Evans L, Hobensack M, Bowles KH (2023). Predicting emergency department visits and hospitalizations for patients with heart failure in home healthcare using a time series risk model. J Am Med Inf Assoc.

[29] Juanes A, Garin N, Mangues MA, Herrera S, Puig M, Faus MJ (2018). Impact of a pharmaceutical care programme for patients with chronic disease initiated at the emergency department on drug-related negative outcomes: a randomised controlled trial. Eur J Hosp Pharm Sci Pract.

[30] Plaza Díaz A, Ruiz Ramos J, Juanes Borrego AM, Blázquez Andión M, Puig Campmany M, Mangues Bafalluy MA (2020). Anticholinergic burden in patients treated for constipation in an emergency department. Emerg.

[31] Brennan JJ, Chan TC, Vilke GM, Killeen JP, Hsia RY, Castillo EM (2017). 211 Emergency Department revisits within three days of an Emergency Department discharge for urinary tract infection among geriatric patients. Ann Emerg Med.

[32] Sparks JA, Vanni KMM, Sparks MA, Xu C, Santacroce LM, Glynn RJ, Ridker PM (2021). Effect of low-dose methotrexate on eGFR and kidney adverse events: a Randomized Clinical Trial. J Am Soc Nephrol.

[33] Sandoval DM, Alarcón GS, Morgan SL (1995). Adverse events in methotrexate-treated rheumatoid arthritis patients. Br J Rheumatol.

[34] Kannan VC, Rasamimanana GN, Novack V, Hassan L, Reynolds TA. The impact of socioeconomic status on emergency department outcome in a low-income country setting: a registry-based analysis. PLoS ONE. 2019;14(10). 10.1371/journal.pone.0223045 PMC6795438 31618277

[35] Guo DY, Chen KH, Chen IC, Lu KY, Lin YC, Hsiao KY (2020). The Association between Emergency Department Revisit and Elderly patients. J Acute Med.

[36] Rodrigues CR, Harrington AR, Murdock N, Holmes JT, Borzadek EZ, Calabro K (2017). Effect of pharmacy-supported transition-of-care interventions on 30-Day readmissions: a systematic review and Meta-analysis. Ann Pharmacother.

[37] Juanes A, Ruíz J, Puig M, Blázquez M, Gilabert A, López L, Baena MI, Guiu JM, Antònia Mangues M. The Effect of the drug-related problems Prevention Bundle on early readmissions in patients from the Emergency Department: a Randomized Clinical Trial. Ann Pharmacother. 2022;10600280221143237. 10.1177/10600280221143237. 10.1177/10600280221143237 36539949

[38] de Hond AAH, Steyerberg EW, van Calster B (2022). Interpreting area under the receiver operating characteristic curve. Lancet Digit Health.

